# Self-Supported Fibrous Sn/SnO_2_@C Nanocomposite as Superior Anode Material for Lithium-Ion Batteries

**DOI:** 10.3390/ma15030919

**Published:** 2022-01-25

**Authors:** Daniele Spada, Pantaleone Bruni, Stefania Ferrari, Benedetta Albini, Pietro Galinetto, Vittorio Berbenni, Alessandro Girella, Chiara Milanese, Marcella Bini

**Affiliations:** 1 Department of Chemistry, University of Pavia, Viale Taramelli 16, 27100 Pavia, Italy; vittorio.berbenni@unipv.it (V.B.); alessandro.girella@unipv.it (A.G.); chiara.milanese@unipv.it (C.M.); bini@unipv.it (M.B.); 2 Center for Colloid and Surface Science, Department of Chemistry, University of Florence, Via della Lastruccia 3, 50019 Sesto Fiorentino, Italy; 3 Department of Pharmacy, University of Chieti-Pescara “G. D’Annunzio”, Via dei Vestini, 66100 Chieti, Italy; pantaleone.bruni@unich.it; 4 National Reference Center for Electrochemical Energy Storage (GISEL)—INSTM, Via G. Giusti 9, 50121 Firenze, Italy; 5 Department of Physics, University of Pavia, Via Bassi 6, 27100 Pavia, Italy; benedetta.albini01@ateneopv.it (B.A.); pietro.galinetto@unipv.it (P.G.)

**Keywords:** tin oxides, carbon nanofibers, anode, LIBs, electrospinning

## Abstract

Low-cost and simple methods are constantly chased in order to produce less expensive lithium-ion batteries (LIBs) while possibly increasing the energy and power density as well as the volumetric capacity in order to boost a rapid decarbonization of the transport sector. Li alloys and tin-carbon composites are promising candidates as anode materials for LIBs both in terms of capacity and cycle life. In the present paper, electrospinning was employed in the preparation of Sn/SnO_x_@C composites, where tin and tin oxides were homogeneously dispersed in a carbonaceous matrix of carbon nanofibers. The resulting self-standing and light electrode showed a greatly enhanced performance compared to a conventional electrode based on the same starting materials that are simply mixed to obtain a slurry then deposited on a Cu foil. Fast kinetics were achieved with more than 90% of the reaction that resulted being surface-controlled, and stable capacities of about 300 mAh/g over 500 cycles were obtained at a current density of 0.5 A/g.

## 1. Introduction

Lithium-ion batteries (LIBs) are extensively employed as power sources in a wide range of applications owing to their high energy densities, coulombic efficiencies and versatility. The past decade has seen tremendous research efforts towards new and improved materials for the development of the next-generation LIBs with high charge capacities/power densities for electric vehicles (EVs), hybrid electric vehicles (HEVs) and so on [1].

Graphite and other carbonaceous materials were, and still are, the first choice as anode in commercial LIBs due to their higher specific capacities and greater negative redox potentials compared with metal oxides, chalcogenides and polymers [2,3]. The maximum lithium uptake through Li^+^ intercalation in crystalline graphite leads to the formation of LiC_6_ (theoretical capacity, TC, 372 mAh/g) and a structural transformation involving the stacking of graphene layers [4].

However, graphitic carbons can co-intercalate propylene carbonate from the electrolyte together with the Li^+^ ions between the graphitic planes causing the graphite to exfoliate and lose capacity [3]. The uniaxial 10% strain along the edge planes can also damage the Solid Electrolyte Interphase (SEI) layer and reduce the cell’s cycle life [5]. In addition, the low Li^+^ intercalation potential of graphite (0.1 V vs. Li/Li^+^), under some circumstances (low temperature, high charging rates etc.), causes the formation of lithium dendrites with a serious impact on the device safety.

Disordered carbons, including soft (fully turbostratic, with a high degree of defectivity) and hard carbons (discrete fragments of few stacked graphene-like sheets in a highly turbostratic layered structure that, at a larger scale, results in voids and pores), can also be lithiated to a certain extent [3,4]. In principle, hard carbons are high-capacity anode materials with good power capability and long life; however, their poor electrical conductivity (disordered carbons are less conductive than graphite) [6] and large irreversible capacity limits their extensive use in LIBs [2].

Among many alternatives to carbonaceous electrodes, the materials based on mechanisms, such as alloying and conversion, have the potential to achieve much higher specific capacities and power densities, which are necessary to take LIBs to the next level, i.e., the next-generation, to enable the clean energy transition by helping to decarbonise transportation and favour a large diffusion of renewable energy technologies [7].

Li-Si and Li-Sn alloys are considered to be promising materials due to their high specific capacities and low operating potential [8,9]. In particular, tin and tin oxides can react reversibly with lithium by combining alloying and conversion reactions with many prospective intrinsic advantages [7]. Specifically, lithium reactions are possible with Sn (TC 992 mAh/g) [10], SnO (TC 876 mAh/g) [11] and SnO_2_ (TC 781 mAh/g) [12] giving an overall lithium storage capacity of 1494 mAh/g.

SnO_x_ + 2xe^−^ + 2xLi^+^ → Sn + xLi_2_O (1)

Sn + ye^−^ + yLi^+^ → Li_y_Sn (0 ≤ y ≤ 4.4) (2)

During the conversion reaction (1), the oxides react to give metallic tin and Li_2_O, and this latter acts as a ‘glue’ to keep the particles of the Sn alloying material together, while also reducing the overall volume change. However, Li_2_O has low electrical conductivity, which leads to a large irreversible capacity and voltage hysteresis. This is why there was a strong belief that the conversion reaction was absolutely irreversible, which also considered the high energy required to obtain SnO_2_ from Sn and Li_2_O (about 600 kJ/mol) [13]. After the conversion reaction, Sn further reacts with Lithium (reaction (2)) up to the terminal phase in the Li-Sn system (Li_22_Sn_5,_ Li/Sn ratio of 4.4) with a huge volume expansion [14].

Recently, it has been demonstrated, through a series of well-designed experiments, that, apart from reactions (1) and (2), the total process of the lithium reaction with SnO_2_ also includes the formation of intermediate phases, namely Li_2_SnO_3_ and Li_8_SnO_6_, with multiple overlapping reactions that are partially reversible [15,16]. The shortcomings of SnO_2_-based anodes are thus related to the low conversion reaction reversibility and the large volume variation (about 300%) during cycling resulting in pulverization and detachment of the active materials from the current collector.

Nano structuration and morphology tailoring are beneficial to the performance of the cell in terms of diffusion length of both Li^+^ and e^−^ in the active material, in alleviating huge volumetric changes and promoting good electrochemical reversibility [17]. Tin oxide can be obtained in different particle shapes and sizes, e.g., nanorods [18], nanosheets [19], nanospheres [20], nanowires [21], nanotubes [22] or nanoflowers [23].

Another effective way to restrain the massive volume change of SnO_2_ is to distribute and encapsulate tin oxide particles in a carbonaceous matrix (graphite layers [24], disordered carbons [25], graphene [26] and carbon nanotubes [27]) acting as a stress-accommodating phase. Carbon is also crucial to improve the electrical contact between the active material and current collector, preventing contact loss due to its mechanical stability. Further, carbon is known not to react with tin and not to form tin carbide [12].

An efficient and scalable method to obtain homogeneous active material dispersion in a flexible carbonaceous matrix is provided by electrospinning [28], which is capable of producing fibrous mats with porous or hollow structures using a fairly simple experimental setup with operational straightforwardness, thus, making it viable for the preparation of innovative electrode materials [29].

The academic research has been very active recently in developing electrodes for energy devices using electrospun carbon nanofibers (CNFs). The current surge in interest for CNFs for this kind of applications has strong roots in the one-dimensional (1D) nanofiber’s distinctive structure that provides an enhanced surface-to-volume ratio and short transport length for ionic transport also contributing to an effective electron transport along the nanofibers in the longitudinal direction [30].

In addition, CNFs usually show high electronic conductivity and good mechanical properties that make them suitable as conductive fillers [31], anodic active materials [32] and conductive supports for cathodic active materials [33]. In looking for cheap and sustainably produced anodes with high-rate capability and cyclic stability, in recent years, electrospun SnO_2_/C nanofibers have been explored with the specific aim of circumventing the partial electrochemical reversibility and the poor electronic conductivity of the intermediate phases formed during cycling in lithium cells [34,35]. Much work has been devoted to studying metal Sn/C nanofibers [29,36,37,38,39,40,41,42].

Despite the recently achieved performance improvements of Sn-based materials, the use of tin in anodes remains very challenging. Quite a few articles have shown how it is difficult to reach the theoretical capacity of Sn and SnO_x_ for a good number of cycles, often reporting values that do not significantly exceed that of graphite, especially when 0 D nanoparticles and commercial materials were used [35,43,44,45]. There is still a long way to go towards the practical application of Sn and SnO_2_-based anodes in commercial LIBs, with the low initial coulombic efficiency a major concern together with the long-term stability.

Thus, in the present work, motivated by the pressing need for robust and enhanced performance electrodes, we employed electrospinning to produce Sn/SnO_2_@carbon composites, used as free-standing electrodes, without the addition of any binder or conductive agent as a strategy to reduce the excessive mechanical stress responsible for the peeling off from the current collector and simultaneously decreasing the total weight for the benefit of the final energy density. The fast and low-cost production process reported herein facilitates the investigation of the performance of Sn/SnO_x_ for energy storage.

The nanofibrous mats were characterized for their structure, morphology and composition using X-ray Diffraction, Raman Spectroscopy, Thermo-Gravimetric Analysis, Scanning Electron Microscopy and Energy Dispersive X-ray Spectroscopy. The electrochemistry involved in the reaction with Lithium was then investigated with Cyclic Voltammetry, Galvanostatic Cycling with Potential Limitation and Electrochemical Impedance Spectroscopy. The pseudocapacitive contribution to the Li storage capacity was also studied.

## 2. Experimental

### 2.1. Sample Preparation

Polyacrilonitrile (PAN, Merck) was dissolved in N,N-Dimethylformamide (DMF, anhydrous, 99.8%, Merck) and magnetically stirred for 18 h at 60 °C to prepare a 9 wt.% PAN solution. Then, SnO_2_ (Aldrich, 99.9%, monophasic crystalline powder, see Supplementary file, Figure S1) was added with vigorous stirring to obtain a homogenous solution at a nominal Sn/PAN loading of 33 wt.%. The solution was then sonicated in a thermostatic bath at 30 °C, alternating 20 min of sonication and 20 min of stirring, three times. Nanofibrous membranes were spun on a custom-built laboratory device as described in previous works [29].

The collector, covered with aluminum foil with the metal needle in a horizontal setting, were positioned at a fixed distance of 22 cm at a height from the base of 18 cm and an inclination angle of 4–5°. The temperature, the relative humidity and the DC voltage applied were kept constant at about 25 °C, 35% and 20–22 kV, respectively.

The flow rate was set at 0.25 mL/h. As a reference, a plain 9 wt.% PAN sample (without the addition of SnO_2_) was electrospun using the same experimental set up at a 0.80 mL/h flow rate and 15 kV DC voltage. The membranes were stabilized in air for 1 h by heating at 5 °C/min up to 280 °C and then calcined in a 50 cc/min flow of Argon/Hydrogen (95:5) by heating at 10 °C/min up to 400 °C and at 5 °C/min to 700 °C with a final isotherm of 3 h.

Some portions of the calcinated samples were ground at 250 rpm in a planetary mill (PM100, Retsch GmbH, Haan, Germany) equipped with ZrO_2_ jars and spheres, alternating 10 min of grinding with 10 min of pause for a total time of 2 h, to obtain small and homogeneous fragments. Thereby, the membranes were ground to a fine, electrostatic powder. In the following, the samples are named C–P (carbon powder), C–S (carbon self-standing), Sn/SnO_2_@C–P (tin/carbon composite powder) and Sn/SnO_2_@C–S (tin/carbon composite self-standing sheet).

### 2.2. Chemical/Physical Characterization

A Bruker D5005 (Karlsruhe, Germany) diffractometer (Cu Kα radiation 40 kV, 40 mA) equipped with a graphite monochromator and scintillation detector was used to collect X-ray powder diffraction (XRD) patterns in air in the following conditions: angular range 15–100°, step size of 0.02° and counting time of 10 s per step using a silicon sample holder with low background. Rietveld refinement was carried out by means of TOPAS 4.0 Bruker software: background coefficients, scale factor, zero error, lattice parameters and crystallite sizes were refined for all the phases, which were also properly quantified.

Room temperature micro-Raman spectra were performed by means of a Labram Dilor spectrometer (Horiba, Japan) equipped with an Olympus (Tokyo, Japan) microscope HS BX40. The Raman signals were excited by the 632.8 nm red light from a 25 mW He–Ne laser. A cooled CCD camera with 2048 pixels was used as a detector also determining a spectral resolution at about 1 cm^−1^. The reported spectra were obtained with typical integration times of about 60 s and averaged over three runs.

The spatial resolution of sampling was around 2 μm diameter due to the focusing of the laser by a 50× objective. Neutral filters with different optical densities were used to irradiate the samples at different light intensities, leading to power density values from 5 × 10^3^ W/cm^2^ to 5 × 10^5^ W/cm^2^. The sample phase homogeneity was verified by mapping the Raman spectra from different regions of each sample. The parameters of the Raman spectra were extracted by using best fitting procedures based on Lorentzian functions, to determine the ratio between the G and D bands of carbon.

A Zeiss EVO MA10 (Carl Zeiss, Oberkochen, Germany) Scanning Electron Microscope (SEM) was used for the morphological study on gold sputtered samples (Secondary Electrons detector). Energy Dispersive X-ray Spectroscopy (EDX) (Oxford Instruments, Wiesbaden, Germany) analysis data (elemental quantification and distribution maps) and SEM images collected with the Back-Scattered Electrons (BSE) (Carl Zeiss, Oberkochen, Germany) detector were obtained on pristine samples (not sputtered). The measurements were performed at 20 kV with a working distance of 8.5 mm.

SEM images at higher magnification were collected with a FEG-SEM Tescan Mira3 XMU, Variable Pressure Field Emission Scanning Electron Microscope (Tescan USA Inc., Warrendale, PA, USA) located at the Arvedi Laboratory, CISRiC, Pavia. The measurements were made on graphite sputtered samples in secondary electrons mode at 20 kV with an In-Beam SE detector at a working distance of 5 mm.

A simultaneous SDT Q600 TA instrument (TA Instruments, New Castle, DE, USA) was employed for the thermal analysis of the samples (Thermo Gravimetric Analysis (TGA) and Differential Scanning Calorimetry (DSC)). All the measurements were performed in an air atmosphere with a heating rate of 5 °C/min from room temperature to 650 °C.

### 2.3. Electrochemical Characterization

Ground samples (powder) and the commercial SnO_2_ (Aldrich, 99.9%) were mixed with a binder, namely PolyVinylidene Fluoride (PVdF, Aldrich, 10 wt.%) and cast onto a Copper foil after preparation of a slurry with N-Methyl Pyrrolidinone (NMP, Aldrich), then maintained overnight at room temperature and dried in a vacuum oven at 100 °C for 1 h. Afterward, the slurry was hot-pressed, and electrodes were cut in form of discs. The carbonized nanofiber and Sn/SnO_2_@C–S mats were punched in discs without any modification and directly inserted in the cell as working electrodes. The mass loading of all kinds of electrodes ranged from 1 to 2 mg/cm^2^.

All the electrodes were stored in a dry box under argon atmosphere (MBraun, Garching bei München, Germany, O_2_ < 1 ppm, H_2_O < 1 ppm) and employed as working electrode for the assembly of Swagelok cells. Li metal was employed as reference and/or counter electrode and a Whatman GF/A disc as the separator, while 1 M LiPF_6_ in a mixture of ethylene carbonate/diethylene carbonate (EC/DEC; 1:1 *v/v*) was the electrolyte. All the electrochemical tests were run in triplicate.

Cyclic voltammetry (CV) was performed with an Autolab PGSTAT30 Eco Chemie, (Metrohm, Utrecht, The Netherlands), from 0.01 to 3 V, for three cycles at a scan rate of 0.1 mV/s. Then, the scan rates were increased to 0.2, 0.5 and 1.0 mV/s for one cycle each.

The same apparatus was also employed for Electrochemical Impedance Spectroscopy (EIS) measurements, from 10^6^ to 10^−2^ Hz, when a sinusoidal excitation signal with an amplitude of 30 mV was applied to the OCV of the cells and after 1 and 10 cycles of galvanostatic charging and discharging. A Neware (Hong-Kong, China) Battery Testing System (BTS-4000) was employed for the Galvanostatic Cycling with Potential Limitation (GCPL), in the same voltage range, applying growing currents to the electrochemical cells (0.1–1 A/g) for 50 cycles or keeping the same current (0.5 A/g) over 500 cycles.

## 3. Results and Discussion

### 3.1. Chemical/Physical Properties

The SnO_x_/C composite nanofibers were manufactured combining electrospinning and heat treatment of the PAN precursor fibers. PAN is undoubtedly one of the most advantageous polymers to fabricate carbon fibers (even commercially), since its high thermal stability enables it to go through a thermo-oxidative stabilization step and a subsequent high temperature carbonization [46]. In fact, it is well-known that the PAN fibers do not collapse during the thermal treatments.

The initial selection of polymers has important consequences on the morphology of the electrospun nanomaterials and also on the mechanical properties of the as-collected and pyrolyzed fibrous mats. PAN especially, as just outlined, gives exceptionally robust porous carbon fibers that eventually give rise to self-supporting non-woven fabric (see Figure S2). The carbonization step was performed under Ar/H_2_ atmosphere (a moderately reducing environment) to promote the formation of metallic tin to obtain Sn/SnO_2_@C samples.

At the same time, we expected to obtain a hydrogen containing soft carbon due to the maximum temperature of the thermal treatment (700 °C) [47]. Furthermore, metallic Sn can be formed in situ through a mechano-chemical reaction by means of high-energy milling [48]. This way, metallic Sn is not only participating to the electrochemical reaction but, in principle, would also contribute to enhancing the electronic conductivity of the nanofibers.

**Figure 1:**
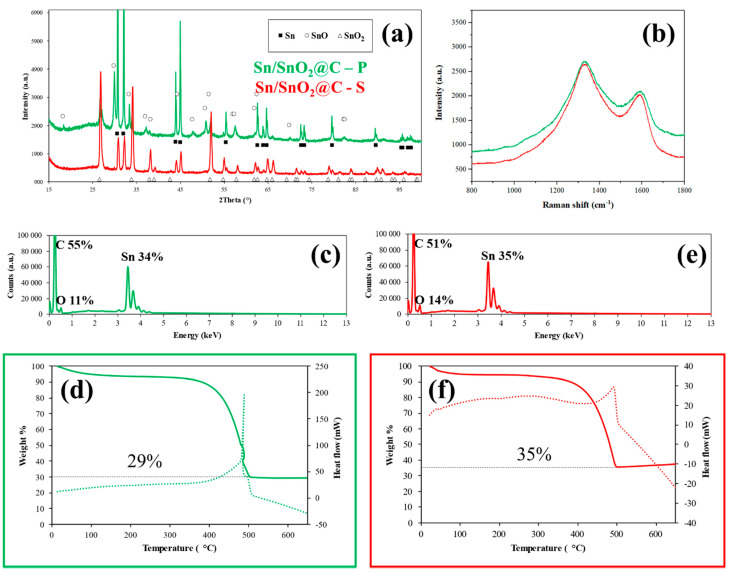
(**a**) XRD patterns, (**b**) Raman spectra, (**c**,**e**) EDX spectra and (**d**,**f**) TGA/DSC curves (full and dotted lines respectively) of Sn/SnO_2_@C–P (green) and Sn/SnO_2_@C–S (red).

The XRD patterns of the self-standing mat obtained by electrospinning and the corresponding ground powder (Figure 1a) revealed, as expected, a blend of different phases, with SnO_2_ (Cassiterite) and tetragonal Sn as the main phases.

In the powdery sample (Sn/SnO_2_@C–P), SnO in a small percentage was also found. The precise quantification of the phases was performed on the powder diffraction patterns by Rietveld refinement, and the results are reported in Table 1. The Goodness of Fit (GoF) values suggested a reliable determination of the parameters.

The Sn/SnO_2_@C -S is mostly constituted by SnO_2_ (ca. 80%) with about 20% of metallic Sn formed by the chemical reduction with Ar/H_2_. The in situ mechano-chemical reaction instead was more effective in reducing Sn(IV), since the primary phase in the Sn/SnO_2_@C–P sample is metal tin (ca. 60%). The heating caused by friction during milling also significantly promoted the crystallization of tin as revealed by XRD (sharper peaks are evident and bigger crystallites are calculated for the ground sample), together with the nucleation and growth of some SnO. Thus, the self-standing carbon mat was richer in SnO_2_, which is about three-times more abundant than in the ground sample, this latter having instead almost three times (precisely 2.85 times) the amount of metallic tin of the former sample.

Carbon could be barely detected in the diffraction patterns because of its low scattering factor and crystallinity but gave distinctive Raman features (Figure 1b). Indeed, all the investigated samples clearly show two broad bands at around 1330 cm^−1^ and 1590 cm^−1^ (the so-called D and G modes, respectively), which are the typical features of disordered carbon materials [49]. The G mode, with E_2g_ symmetry, is due to in-plane bond-stretching motion of pairs of C sp^2^ atoms in single crystals of graphite, while the D mode is a breathing mode of A_1g_ symmetry involving phonons near the K zone boundary, thus, being disorder-induced. The vibrations responsible for the D mode are frequency depending, with resonance effect and its intensity scales inversely with grain dimensions. The broadening affecting both G and D modes and their relative ratio (G/D, see Figure S3) indicate a higher degree of disorder comparable with amorphous carbon and, in this behavior, a role is surely played by the nanometer sized crystallites and the highly turbostratic structure [50,51].

The corresponding Raman spectra and XRD patterns of the Sn-free carbon nanofiber mats are typical of turbostratic, little crystalline soft/hard carbons (Figure S4) [52], which completely decomposed during TGA/DSC measurements. The high interconnectivity of the PAN nanofibers and the low temperature of the thermal treatment (relatively to graphitization) could have led to a high amount of defectivity in the resulting carbon [47].

The chemical composition of the samples was verified by EDX and TGA/DSC analyses (Figure 1c–f). The spectroscopic quantification (Figure 1c,e) yielded an estimated amount of 34 and 35 wt.% of Sn atoms in the Sn/SnO_2_@C–P and Sn/SnO_2_@C–S samples, in very good agreement with the stoichiometry of the synthesis. The thermogravimetric analysis (Figure 1d,f) revealed a small mass loss, completed at around 100 °C, probably due to adsorbed humidity, followed by the exothermic combustion of carbon in air starting from 320 °C. After the combustion, the remaining of about 29% and 35% wt.%, which is the residual tin, gave confirmation of the aforementioned results.

**Figure 2:**
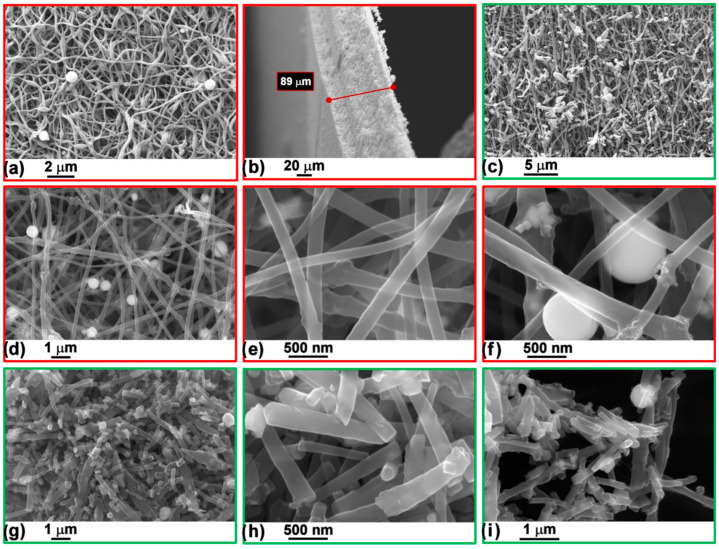
SEM (**a**–**c**) and FE-SEM (**d**–**i**) images at different magnifications collected on Sn/SnO_2_@C–S (**a**,**b**,**d**–**f**) and Sn/SnO_2_@C–P (**c**,**g**–**i**). A cross-section image of the self-supporting sample is also shown (**b**).

The morphological analysis of the Sn/SnO_2_@C–S composite showed the typical appearance of electrospun fiber mats (Figure 2a): a highly interconnected network of micrometer long nanofibers having sub micrometer diameters.

A few spherical beads can be seen on the surface of the fibers, which seem to have slightly higher concentration of Sn (Figure S5—BSE images). These granules might have been generated by Sn nanoparticles coalescence during the thermal treatment; however, in general, tin atoms appeared to be homogeneously distributed all over the sample (Figure S5—EDX maps).

The Sn-free PAN-derived carbon nanofibers mat (Figure S4) showed the same features, i.e., a texture of intimately tangled fibers. This sample has no beads, confirming that tin is responsible for their formation. Figure 2b shows the cross-section of the nanofiber mat, which is around 90 μm. As a consequence of grinding the mat in the high-energy ball miller, the length of the fibers is reduced in both the Sn/SnO_2_@C–P and Sn-free samples (Figure 2c and Figure S4), which are mostly constituted by fragmented, shorter (1–5 μm long) and less interconnected cylindrical nano-fibres, to give an actual fine powder.

Higher magnifications images (Figure 2d–i) allowed a better inspection of the spherical beads (500 nm diameter) and fibers, which were determined to be 200 nm wide (slightly larger for the milled sample) and did not show any evidence of pores on their surface, as a consequence of CO_2_ evolved during the thermal treatment of PAN.

### 3.2. Electrochemistry

The lithium storage properties of the Sn/SnO_2_@C samples were evaluated in half-cell configuration. Cyclic voltammetry (CV) can provide useful information about the thermodynamics and kinetics of Lithium intercalation reactions, which indeed define the shape of the voltammograms, so that, through suitable experiments, some valuable parameters can be extracted.

**Figure 3:**
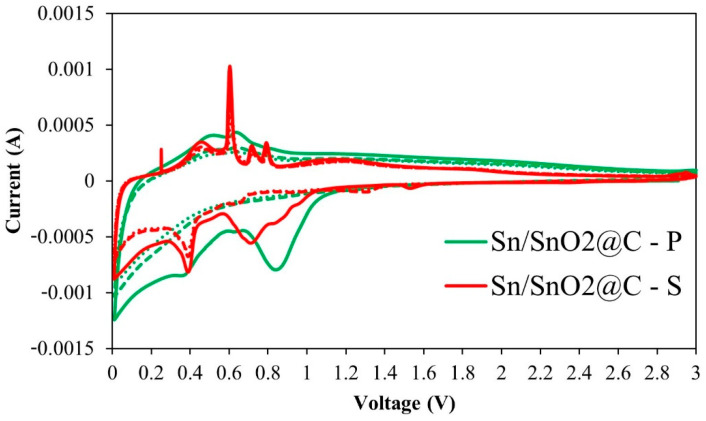
Voltammograms performed at 0.1 mV/s of the first three cycles on Sn/SnO_2_@C composites. The first cycle is depicted with a solid line.

The voltammograms of Sn/SnO_2_@C samples are reported in Figure 3 (see also Figure S6 for the CV of the commercial SnO_2_). Many redox peaks can be clearly observed which are indication of the multiple reactions taking place during the lithiation/delithiation processes. In the voltage range below 1 V, the main features appeared related to (partially) reversible and irreversible reactions. During the first scan, a large cathodic peak is observed with a maximum at about 0.71 V (Sn/SnO_2_@C–S) and 0.85 V (Sn/SnO_2_@C–P), ascribed to some overlapping reactions including the formation of the solid electrolyte interphase (SEI) layer due to the EC/DEC reduction and the conversion reaction of SnO_2_ to Sn and Li_2_O.

In addition, as recently reported, we cannot exclude the formation of some intermediate Li_x_SnO_y_ phase by insertion of Li^+^ in SnO_2_ at about this voltage and before the conversion reaction is completed [15,16]. Minor, but distinct peaks, can be seen for the Sn/SnO_2_@C–S sample indicating the reduction of SnO_2_ to SnO and to metallic Sn. These peaks are not distinguishable for the powdery sample, for which only the broader peak due to SEI formation at the carbon nanofibers surface is seen, which extends to lower voltages for both the mat and the powder likely for kinetic reasons.

Despite the SEI layer being formed in both samples, some morphological and chemical characteristics, such as the presence of defects and the surface-to-volume ratio, can govern the extent of the SEI formation, which indeed appears different by comparing these samples. In addition, in the Sn/SnO_2_@C–P sample the SnO_2_ percentage is lower than for the self-supporting electrode, which might also account for the absence of clearly visible conversion peaks that are, in any case, of very low intensity whenever present. The other cathodic peaks at lower potentials are related to the formation of SnLi_x_ alloys with increasing Li:Sn ratio, up to Li_4.4_Sn.

In the following cycles, the voltammogram of the P sample showed marked similarities with the carbon samples (Figure S7), suggesting that the alloying reaction might be less efficient due to the well-known issue of Sn particle coarsening. Sn coarsening produces larger particles that cannot fully alloy with Li during the discharge originating what is called “dead” Sn. The Sn/SnO_2_@C–S sample (Figure 3) showed instead a sharp and stable peak at 0.39 V corresponding to the alloying reaction, which suggests a stable morphology for the metallic Sn particles.

Well-defined features are also detected in the anodic scan, where the multiple peaks at 0.47, 0.61, 0.72 and 0.79 V are attributed to the de-alloying reactions. Analogous, but much broader, peaks are detected in the anodic scan of the P sample and similarly of the commercial SnO_2_ (Figure S6). When the potential window is large enough, above 1.2 V the metallic Sn, which has been obtained by delithiation of the alloys, is oxidized to obtain SnO_2_.

The differences in the voltammograms can be mainly attributed to the different morphological characteristics of the samples, since the general electrochemical response should be similar after the first reduction (lithiation), regardless of the initial composition in terms of Sn phases. However, for the self-supporting electrode, the nanofibers were not broken and formed a network suitable to enhance the electron conductivity, so that the reversibility of the electrochemical reaction was improved compared to that of the P and commercial samples.

Well-interconnected webs are much more effective than the aggregates of cracked nanorods in tolerating the large volume changes that Sn particles undergo upon continuous cycling. Furthermore, the homogenous dispersion of tin into the carbonaceous matrix provided a high-conducting substrate that could finally enhance the rate capability of the material.

**Figure 4:**
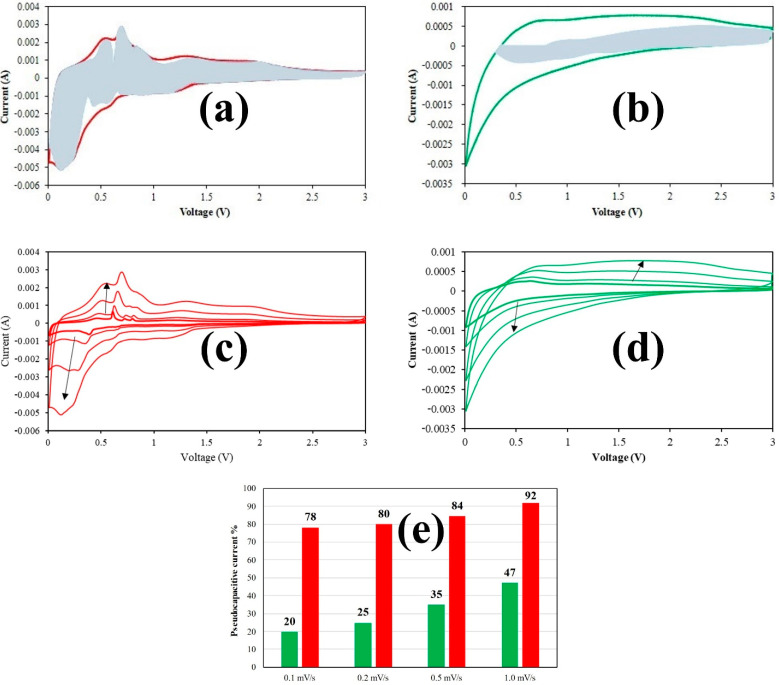
Voltammograms at 1.0 mV/s with pseudocapacitive currents evidenced in light grey for Sn/SnO_2_@C–S (**a**) and Sn/SnO_2_@C–P **(b**) and corresponding voltammograms at all the sweep rates (**c**,**d**). Histogram of the total contribution of the pseudocapacitive current at all sweep rates (**e**).

Figure 4 shows the voltammograms at different sweep rates (c,d) and the corresponding histograms (e) quantifying the pseudocapacitive contribution to the experimentally recorded current.

A material is said to be pseudocapacitive when reversible redox reactions occur at (or near) the surface in contact with an electrolyte, or when these reactions are not limited by solid-state ion diffusion. This feature leads to a high energy density at high charge–discharge rates and can be an intrinsic or extrinsic property. Extrinsic materials do not exhibit pseudocapacitance in the bulk state due to phase transformations during ion storage; an increase in their surface area through nanostructuring normally leads to improving the high-rate behavior due to a decrease in the diffusion distances [53].

In a CV experiment, the timescale is controlled by the sweep rate (*v*, mV/s). The current response to an applied sweep rate will vary depending on whether the redox reaction is diffusion-controlled or surface-controlled (capacitive). For a redox reaction limited by semi-infinite linear diffusion, the current response varies with *v*^1/2^; for a capacitive process, the current varies directly with *v*. Therefore, for any material the following general relationship, Equation (3), may be written for the current at a certain potential:

*i*(V) = k_1_ v^1/2^ + k_2_ v (3)

Solving for the values of k_1_ and k_2_ at each potential allows for the separation of the diffusion and capacitive currents [53,54].

Since the pseudocapacitance of tin and tin oxides is extrinsic, a higher pseudocapacitive contribution can be considered as an indication of higher electrode surface. The self-standing composite (and the corresponding carbon sample, Figure S7) show higher pseudocapacitive contributions with respect to their powder counterpart, up to 90% at 1 mV/s, demonstrating that electrospinning can be very effective in improving the rate capability of electrode materials, which is especially advantageous for conversion-alloying reactions.

The increase in the sweep rate (up to 10 times) did not cause the shape of the voltammogram of the Sn/SnO_2_@C–S sample to change significantly (Figure 4c). On the contrary, most of the current recorded in the anodic scan for the powder sample (Figure 4d) is shifted to higher potentials, which is detrimental for anode materials. As evidenced by the histogram plot (Figure 4e) the capacitive-reaction processes are significant at all sweep-rates for the Sn/SnO_2_@C–S sample with the second anodic peak always higher than the first anodic peak, this latter being likely related to diffusion reactions, as previously reported [55].

**Figure 5:**
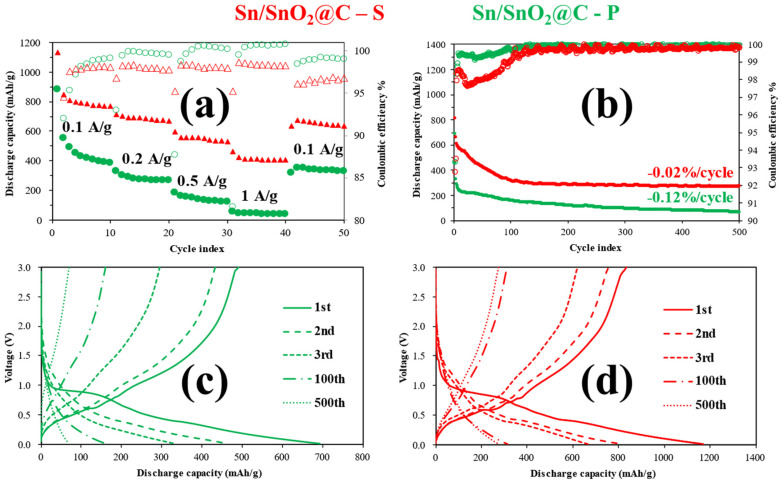
Rate capability (**a**), long term tests performed at 0.5 A/g (**b**) and corresponding voltage profiles of Sn/SnO_2_@C–P (**c**) and Sn/SnO_2_@C–S (**d**). In the upper graphs, the empty markers refer to the coulombic efficiency (right axis), while the filled ones refer to the specific capacity (left axis).

The high pseudocapacitance is at the base of the high-rate performance of Sn/SnO_2_@C–S. Figure 5a shows the results of the rate capability tests. The self-standing sample outperformed the powder sample by hundreds of mAh/g at every C-rate (see Table S1 for some representative cycles) as well as the commercial SnO_2_ (Figure S9), which showed a similar response as the Sn/SnO_2_@C–P sample. The high electrode area and good interconnection of the carbon nanofibers enhanced the electrochemical reactivity and reversibility of the active material, which achieved a superior performance, especially at higher C-rates.

The first cycle coulombic efficiency of Sn/SnO_2_@C–S was 77%, which is much higher than the 52% average value for bulk SnO_2_ gathered over many published articles [13]. The capacity loss (quantified by the coulombic efficiency) in the first few cycles is ascribed to the partially reversible and irreversible reactions involving the active materials and the SEI formation (evidenced also in the voltammograms). The coulombic efficiency rapidly reached values higher than 97% in the following cycles.

Long-term stability tests (Figure 5b) confirmed the improved performance of Sn/SnO_2_@C–S over 500 cycles: a stable (−0.02% loss/cycle) capacity of 275 mAh/g is obtained at 0.5 A/g, with a coulombic efficiency of 99.8%. As with the rate capability tests, the commercial SnO_2_ (Figure S9) had a comparable performance to the ball-milled sample; the specific capacity decreased gradually up to 100 cycles, and the capacity retention was poor for both samples. The voltage profiles (Figure 5d) did not change after the 100th cycle for the self-supporting electrode but became progressively linear for the P sample (Figure 5c); this marked difference is likely due to the flexible and more conductive carbonaceous matrix in the former sample, which is also beneficial for buffering volume changes.

**Figure 6:**
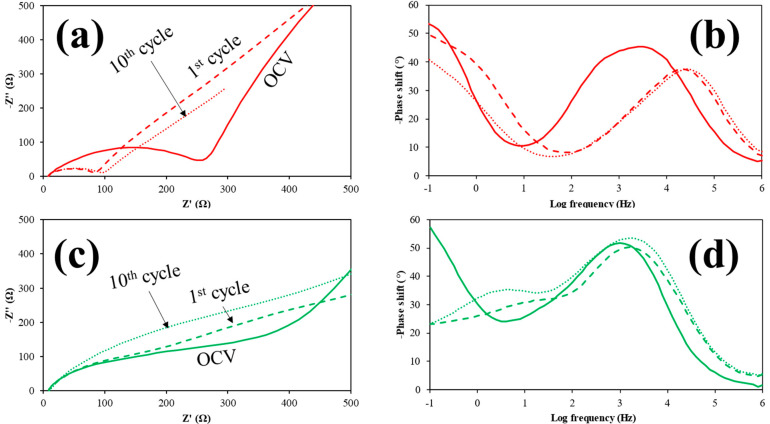
Nyquist plot and corresponding Bode phase plot of Sn/SnO_2_@C–S (**a**,**b**) and Sn/SnO_2_@C–P (**c**,**d**).

To obtain further insights into the behavior of the Sn/SnO_2_@C–P sample, EIS spectra were collected. The Nyquist plots (Figure 6a,c) showed lower resistance from the Open Circuit Voltage onwards for the self-standing sample. The higher resistance to the charge transfer of electrons (which occurs at high frequencies, i.e., 10^3−4^ Hz, see Figure 6b,d) of the powder sample (Figure 6c) can be attributed to the presence of the PVDF binder, which is insulating, and to the loss of interconnection among the carbon nanofibers.

After the first cycle, the impedance spectrum of the self-standing sample showed a significant decrease in the resistance (Figure 6a), which was not detected in the powder sample (Figure 6c) and is consistent with a low reversibility of the conversion reaction of SnO_2_ (semiconductor, the main phase in the S sample) to Sn (metal, the main phase in the P sample). This decrease in the resistance is accompanied by a significant shift of the charge transfer of electrons to higher frequencies (more than one order of magnitude, see Figure 6b), again not evidenced in the powder sample (Figure 6d). Thus, the main origin of the constant and higher resistance offered by the P sample might be due to the insulating PVdF used to prepare the electrode.

Finally, ex situ SEM images were collected on the electrodes after disassembling the cells (Figure S9). The volumetric expansion and contraction during cycling did not pulverize the electrodes as has been often reported for conversion-alloying materials. It is likely that the rod-like morphology significantly tailored the mechanical stress that the electrodes underwent upon the lithiation/delithiation processes.

## 4. Conclusions

Sn/SnO_2_@C composites were successfully electrospun to obtain a self-standing electrode that was compared with a coated-dried classical one prepared after ball milling the original nanofibers to obtain a fine powder. In the composites, the turbostratic carbonaceous matrix revealed a web-like morphology, and the dispersion of tin was homogeneous within the samples. Crystalline Sn, SnO and SnO_2_ were all detected in the ball-milled sample, and the main phase metallic Sn was produced due to the mechano-chemical route.

The self-standing electrode only contained Sn and SnO_2_ with this latter as the main phase. Each of the Sn phases offers specific advantages in the electrochemical reactions with lithium. The self-standing electrode showed an enhanced reactivity with stable current peaks over the cycles and at different sweep rates (>90% of the current showed surface-controlled kinetics). In the rate capability test, it also outperformed, by hundreds of mAh/g, the conventional electrode made by the ball-milled sample, thus, maintaining a good performance at higher current densities.

After 500 cycles of charge and discharge, a stable (0.02% loss/cycle) and good capacity of 275 mAh/g was reached for the Sn/SnO_2_@C–S sample. The electrodes did not pulverize after prolonged cycling, and the resistance offered by the self-standing electrode to the charge transfer of electrons was significantly lower than that of the classical electrode, likely due to the absence of an insulating binder.

The easy, fast and low-cost method here reported enabled a homogeneous distribution of Sn and SnO_2_ in the CNFs matrix, which is essential, together with the self-standing design to develop promising and more performing anodes, for LIBs to achieve a green transition in the energy sector.

## Figures and Tables

**Table 1:** Quantitative Phase Analysis performed by the Rietveld refinement on the XRD patterns of the tin/carbon composites. Calculated crystallite sizes and R_wp_/GOF values are also reported.

	Sn (wt.%)	Cry. Size (nm)	SnO (wt.%)	Cry. Size (nm)	SnO_2_ (wt.%)	Cry. Size (nm)	R_wp_/GOF
Sn/SnO_2_@C–P	62.28 (46)	120.9 (12)	12.29 (26)	33.53 (65)	25.43 (23)	45.9 (19)	5.45/1.39
Sn/SnO_2_@C–S	21.80 (19)	44.37 (86)	-	-	78.20 (19)	40.50 (37)	9.57/1.74

## Data Availability

The data are available on request.

## Supplementary Materials

The following supporting information can be downloaded at: https://www.mdpi.com/article/10.3390/ma15030919/s1, Figure S1: XRD pattern and SEM images of commercial SnO_2_; Figure S2: Picture of the self-supporting non-woven fabric; Figure S3: Example of fitting of Raman spectrum of C–S sample and ratios of D and G areas; Figure S4: XRD patterns, TGA/DSC curves, Raman spectra and SEM images of carbonized PAN samples; Figure S5: SEM images, corresponding EDX maps of Sn and BSE images for SnO_x_/C samples; Figure S6: Voltammograms of commercial SnO_2_; Figure S7: CV of carbon samples, histograms of the pseudocapacitive current and voltammogram of C–P; Figure S8: Rate capability and long-term test on commercial SnO_2_; Figure S9: Ex situ SEM images collected on the electrodes of pristine; Table S1: Discharge capacity and coulombic efficiency values for some representative cycles of the rate capability tests on Sn/SnO_2_@C samples.
