# Supplementary material for: Self-Supported Fibrous Sn/SnO2@C Nanocomposite as Superior Anode Material for Lithium-Ion Batteries
Source: Materials (Basel). 2022 Jan 25;15(3):919. doi: 10.3390/ma15030919 (PMC8839326; doi:10.3390/ma15030919)
Supplement: Supplementary file 1 [file materials-15-00919-s001.zip › materials-1535920-supplementary.pdf]

materials

Supplementary

Self-Supported Fibrous Sn/SnO_2@C Nanocomposite as Superior Anode Material for Lithium-Ion Batteries

Daniele Spada, Pantaleone Bruni, Stefania Ferrari, Benedetta Albini, Pietro Galinetto, Vittorio Berbenni, Alessandro Girella, Chiara Milanese and Marcella Bini

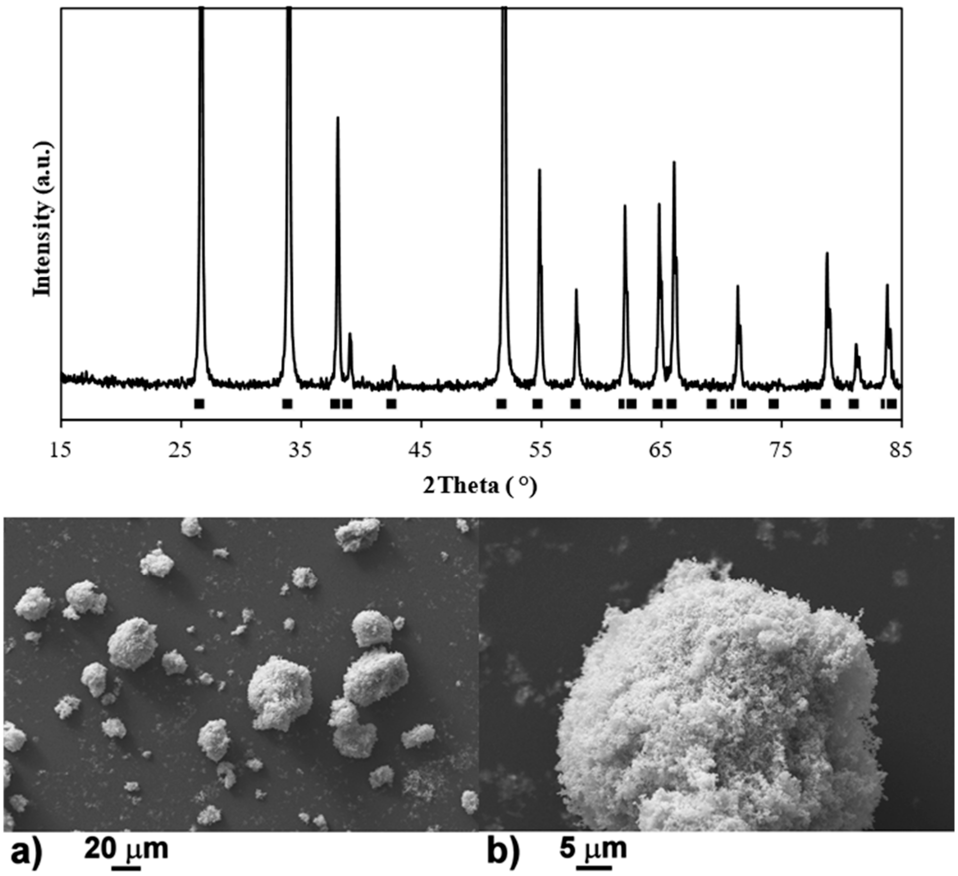

Figure S1. XRD pattern, with reflections of Cassiterite (squares), and SEM images at low (**a**) and high (**b**) magnification of commercial SnO_2.

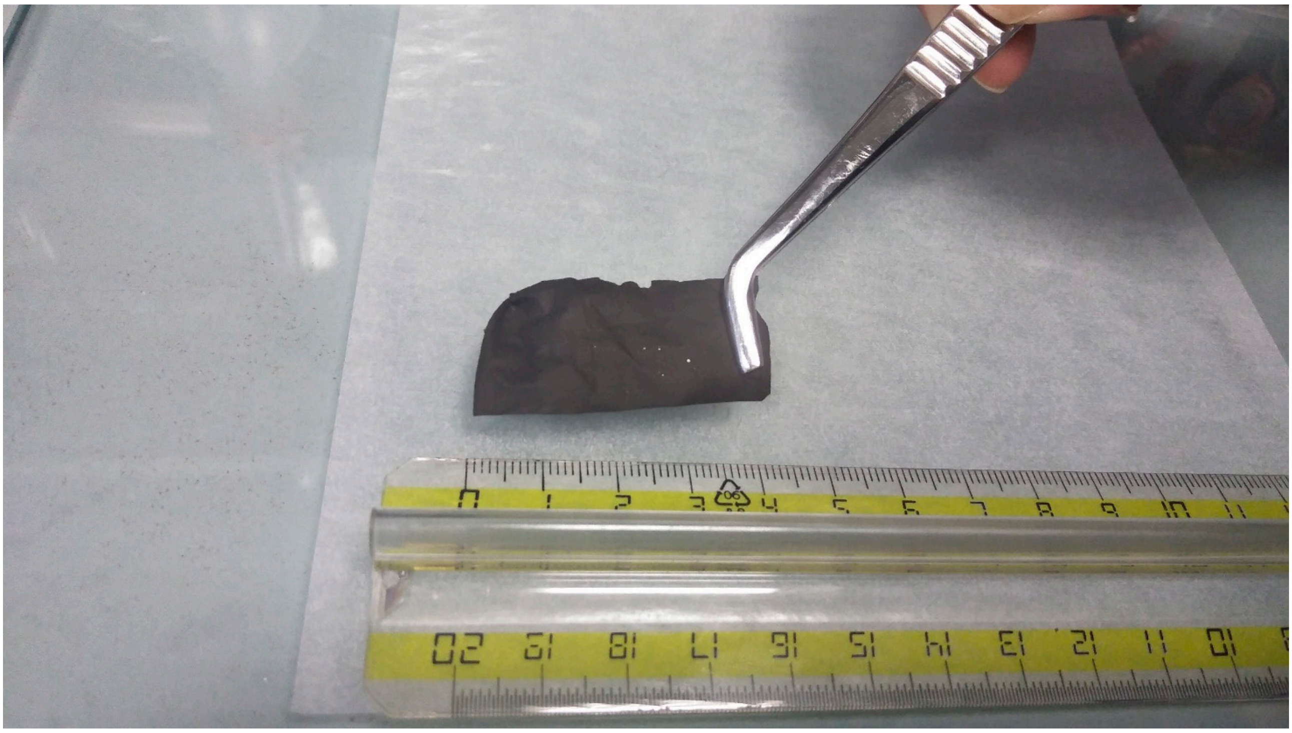

Figure S2. Picture of the self-supporting non-woven fabric, close to a ruler (the scale is in cm).

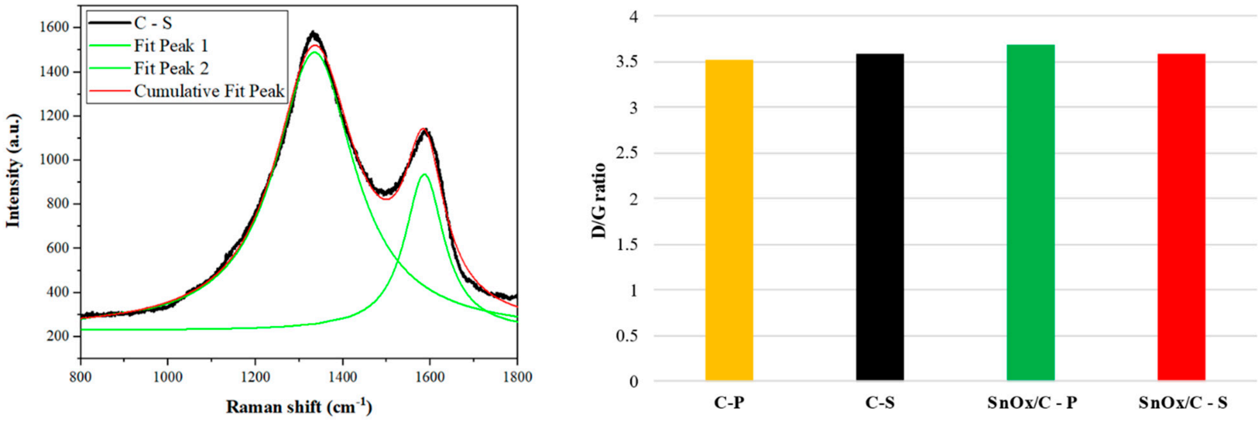

Figure S3. Example of fitting of Raman spectrum of C–S and corresponding ratios between the areas of the D and G bands for all the samples.

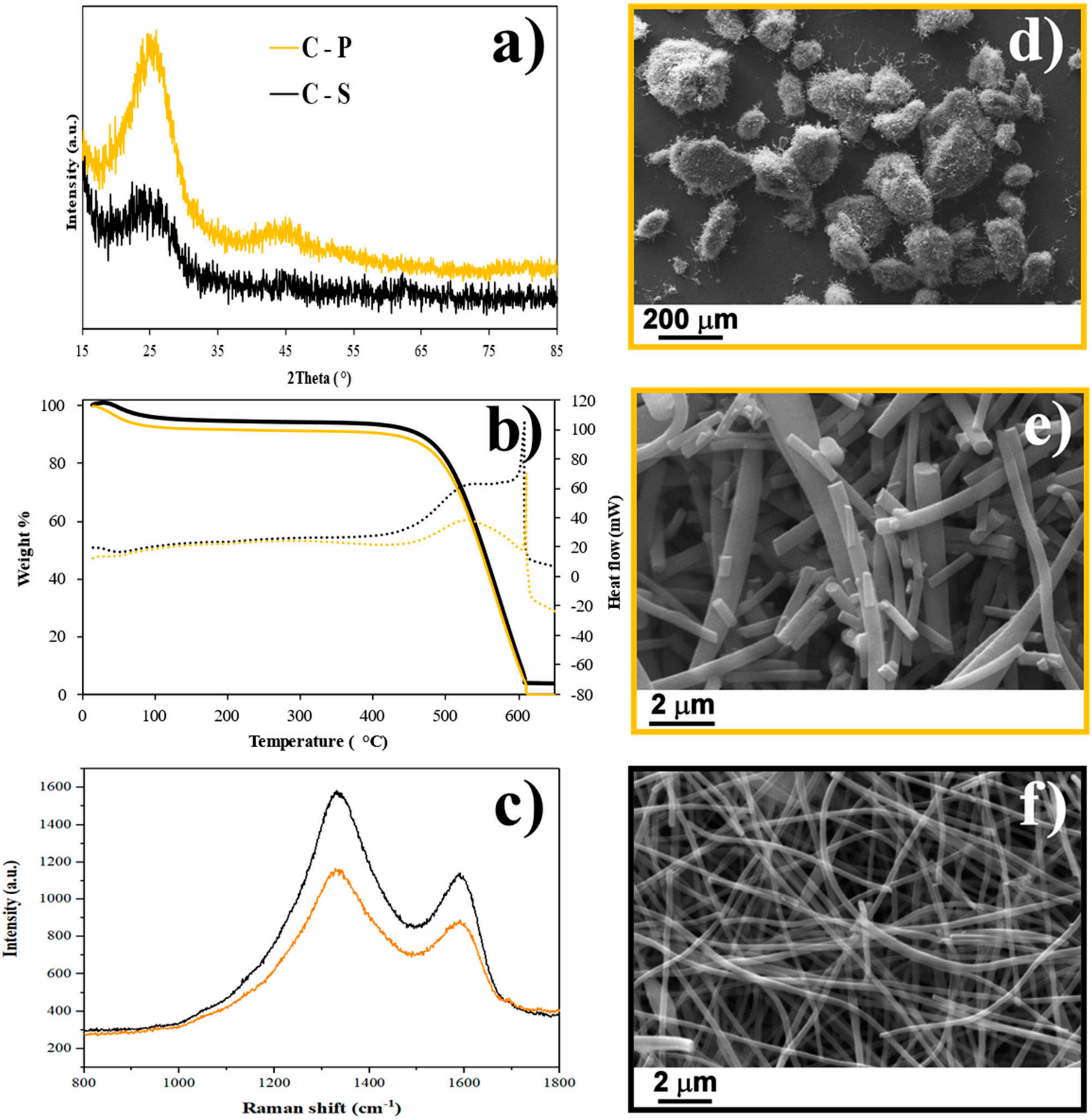

Figure S4. XRD patterns (**a**), TGA (full lines)/DSC (dotted lines) curves (**b**), Raman spectra (**c**) and SEM images (**d**–**f**) of carbonized PAN samples (orange C–P, black C–S).

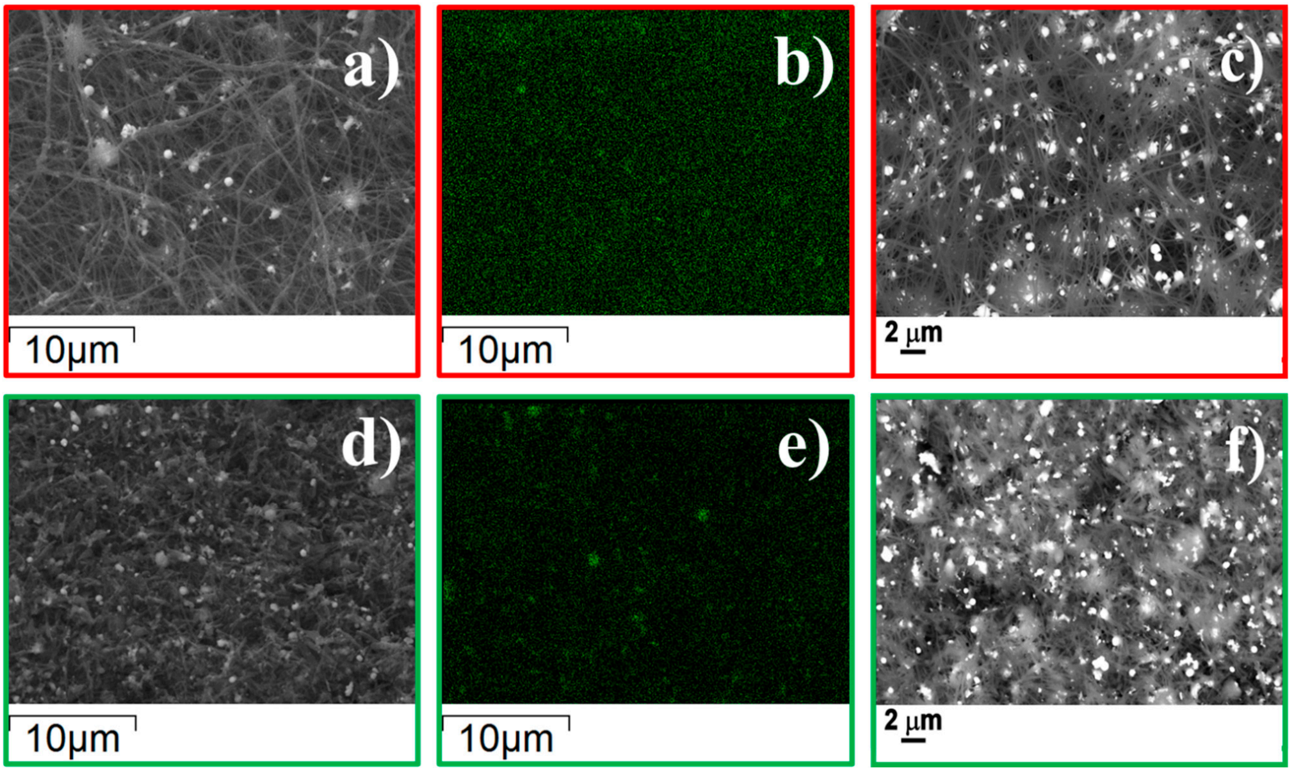

Figure S5. SEM images from secondary electrons, corresponding EDX maps of Sn and backscattered electrons SEM images for SnOx/C–S (**a**–**c**) and SnOx/C–P (**d**–**f**) respectively.

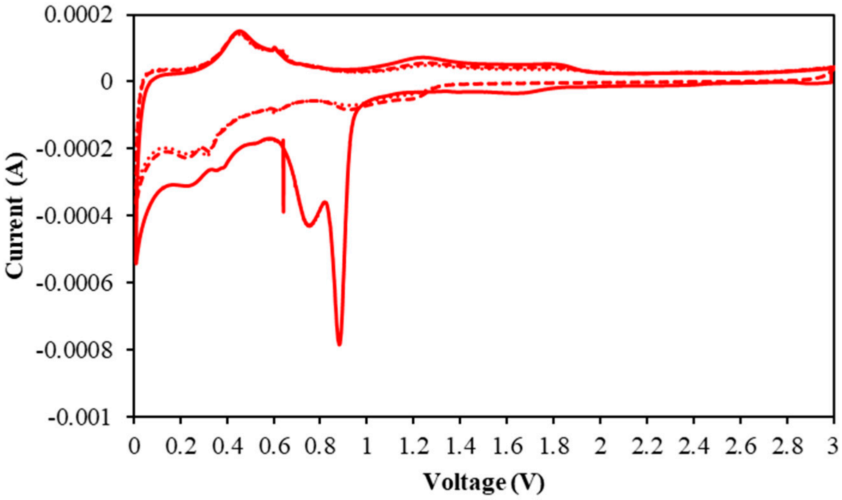

Figure S6. Voltammograms of the first three cycles performed at 0.1 mV/s on commercial SnO_2. The first cycle is depicted with a solid line.

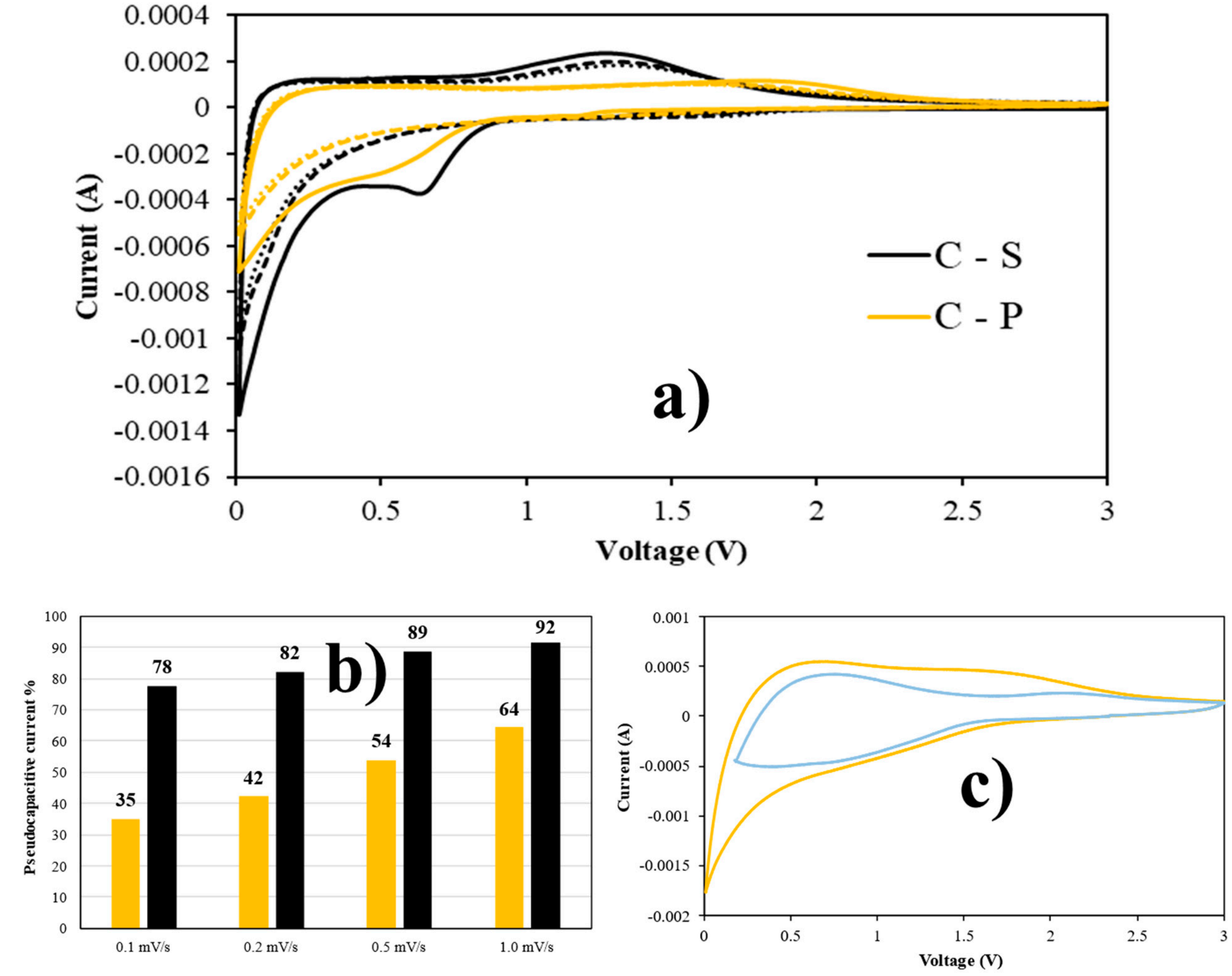

Figure S7. First three cycles of cyclic voltammetry of carbon samples (**a**). The first cycle is depicted with a full line. Histograms of the pseudocapacitive current (**b**) and voltammogram of C-P at 1.0 mV/s (**c**) with pseudocapacitive current in light blue.

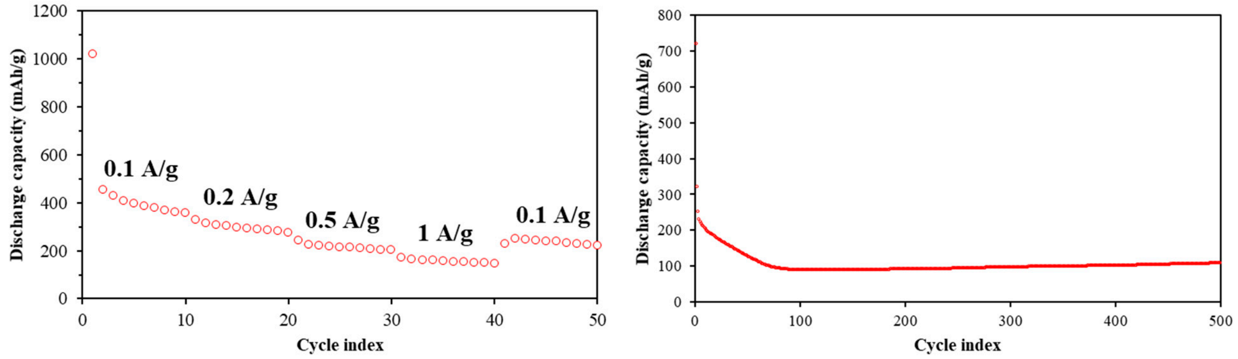

Figure S8. Rate capability (on the left) and long-term test (on the right) performed at 0.5 A/g on commercial SnO_2

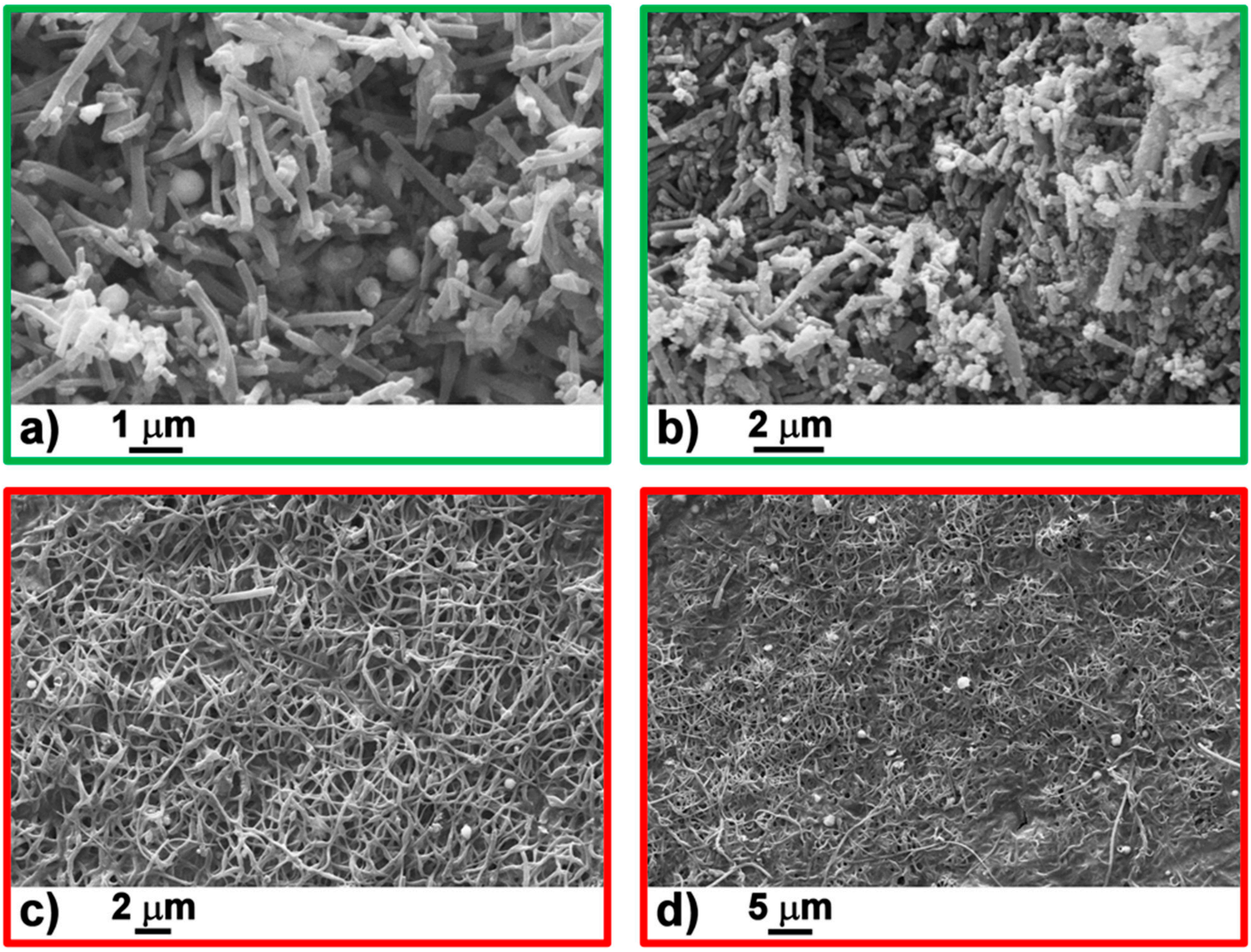

Figure S9. Ex situ SEM images collected on the electrodes of pristine and post-mortem Sn/SnO_2@C–P (**a**,**b**) and Sn/SnO_2@C–S (**c**,**d**) respectively.

Table S1. Discharge capacity and corresponding coulombic efficiency values for some representative cycles of the rate capability tests on Sn/SnO_2@C–P and Sn/SnO_2@C–S.

		Sn/SnO_2@C - P		Sn/SnO_2@C - S	
**Current density (A/g)**	**Cycle index**	**Capacity (mAh/g)**	**Coulombic efficiency %**	**Capacity (mAh/g)**	**Coulombic efficiency %**
**0.1**	1	881	68.0	1131	76.8
	2	554	92.0	850	94.6
	10	383	99.1	768	98.1
**0.2**	12	302	99.4	702	98.3
	20	266	99.5	669	97.8
**0.5**	22	161	98.8	560	98.3
	30	123	100.2	531	98.0
**1**	32	45	99.6	424	98.7
	40	35	100.8	408	98.3
**0.1**	42	351	98.5	673	96.2
	50	327	99.1	637	96.9
